# Early Troponin T and Prediction of Potentially Correctable In-Hospital Complications after Coronary Artery Bypass Grafting Surgery

**DOI:** 10.1371/journal.pone.0074241

**Published:** 2013-09-06

**Authors:** Volkhard Göber, Andreas Hohl, Brigitta Gahl, Florian Dick, Verena Eigenmann, Thierry P. Carrel, Hendrik T. Tevaearai

**Affiliations:** Department of Cardiovascular Surgery, Bern University Hospital (Inselspital) and University of Bern, Bern, Switzerland; S.G.Battista Hospital, Italy

## Abstract

**Background:**

Peak levels of troponin T (TnT) reliably predict morbidity and mortality after cardiac surgery. However, the therapeutic window to manage CABG-related in-hospital complications may close before the peak is reached. We investigated whether early TnT levels correlate as well with complications after coronary artery bypass grafting (CABG) surgery.

**Methods:**

A 12 month consecutive series of patients undergoing elective isolated CABG procedures (mini-extra-corporeal circuit, Cardioplegic arrest) was analyzed. Logistic regression modeling was used to investigate whether TnT levels 6 to 8 hours after surgery were independently associated with in-hospital complications (either post-operative myocardial infarction, stroke, new-onset renal insufficiency, intensive care unit (ICU) readmission, prolonged ICU stay (>48 hours), prolonged need for vasopressors (>24 hours), resuscitation or death).

**Results:**

A total of 290 patients, including 36 patients with complications, was analyzed. Early TnT levels (odds ratio (OR): 6.8, 95% confidence interval (CI): 2.2-21.4, P=.001), logistic EuroSCORE (OR: 1.2, 95%CI: 1.0-1.3, P=.007) and the need for vasopressors during the first 6 postoperative hours (OR: 2.7, 95%CI: 1.0-7.1, P=.05) were independently associated with the risk of complications. With consideration of vasopressor use during the first 6 postoperative hours, the sum of specificity (0.958) and sensitivity (0.417) of TnT for subsequent complications was highest at a TnT cut-off value of 0.8 ng/mL.

**Conclusion:**

Early TnT levels may be useful to guide ICU management of CABG patients. They predict clinically relevant complications within a potential therapeutic window, particularly in patients requiring vasopressors during the first postoperative hours, although with only moderate sensitivity.

## Introduction

Modern immunoassays are able to detect myocardial troponin T (TnT) in peripheral blood serum with such a high sensitivity that acute coronary syndromes can be diagnosed early enough to improve the patients’ prognosis drastically [1,2]. For the assessment of cardiac perfusion after cardiac surgery, however, such assay sensitivity does not represent an advantage because troponins, as well as other cardiac ischemia markers, are almost always elevated after interventions on the heart [3]. The direct surgical injury, the damage associated with an insufficient cardioplegic protection or following blood reperfusion, and the lesions due to electrical defibrillation are all mechanisms that may affect the myocardial integrity [4]. Therefore, immediate post-operative TnT may be seen as a surrogate parameter globally reflecting how the heart tolerated the intervention. However, post-operative elevation of TnT does not necessarily imply ongoing myocardial infarction (MI). Indeed, the definition of a precise TnT cut-off value to characterize a postoperative myocardial infarction has been difficult to determine, but would be clinically important [5–8].

Postoperative levels of TnT are known to correlate with early or mid-term morbidity and mortality [6,9], possibly even with long-term survival [10–12], and it seems that TnT levels at 48 hours post-surgery have the highest prognostic power [6,10]. Although useful as an epidemiological predictor of postoperative complications [5,13,14], late assessment of TnT values is of limited individual screening interest because the therapeutic window for perioperative acute coronary syndrome closes just a few hours after myocardial reperfusion.

The present study aimed to explore whether early TnT values after coronary artery bypass grafting (CABG) predicted clinically relevant in-hospital complications. If so, this marker might be useful to guide intensive care unit (ICU) management toward improved hospital outcome.

## Materials and Methods

The study investigated a consecutive series of patients undergoing isolated CABG procedures at a single tertiary referral cardiac surgery unit in Switzerland between January 1^st^ and December 31^st^ 2008. Patients were identified from a prospectively maintained institutional registry (Intellect 1.6.5, Dendrite Clinical Systems, Henley-on-Thames, UK). Since isolated CABG procedures were performed almost exclusively under mini ECC (MECC), those using a regular ECC as well as beating heart procedures were excluded to warrant a homogenous study population. Patients with a recent myocardial infarction (occurring less than 7 days before CABG) were excluded as well, to avoid elevated TnT levels before surgery. However, preoperative TnT levels were not routinely measured in this patient population. All patients provided informed written consent for subsequent anonymized observational analyses of their data at the time of surgery, and the institutional review board of the University Hospital of Berne approved the observational design of the present investigation as part of our regular institutional quality audit.

### Study design and outcome measures

Analytic strategy was agreed before data were inspected. The main analysis focused on independent correlations between early postoperative TnT levels after CABG and the occurrence of a composite of clinically relevant in-hospital complications which included MI, stroke, new-onset renal insufficiency, ICU stay of more than 48 hours or readmission to the ICU, need for inotropes or vasopressors for more than 24 hours, resuscitation and death. Indication of inotropes and vasopressors were signs of insufficient tissue perfusion despite fluid resuscitation and presumed sufficient (norepinephrine) or insufficient cardiac output (epinephrine, dobutamine).

Secondary analyses aimed at defining a TnT threshold level with optimized predictive value and explored whether early TnT levels were independently associated with long-term prognosis.

### Surgery and Anesthesia

All procedures were performed via full sternotomy and under moderate systemic hypothermia (32°C) using a MECC system [15] (Jostra Hirlingen, Germany) and a single shot mini-cardioplegia (100 ml Cardioplexol^TM^, Laboratorium Dr G. Bichsel AG, Unterseen, Switzerland). Cardioplexol^TM^ is a crystalloid solution, which is injected directly into the aortic root. Cardiac arrest characteristically occurs within 5-8 seconds allowing the surgeon to proceed without any delay to the procedure. Typically, the LIMA, the left radialis artery and/or a segment of the great saphenous vein were used as bypass conduits. Anesthetic protocol was standardized for all patients and included the use of fentanyl, midazolam and isoflurane.

### Predictor variables and potential confounding factors

The main predictor variable was the early postoperative TnT level. TnT, creation kinase (CK) and CK-MB levels were all assessed at least twice after CABG during postoperative routine blood sampling which was scheduled at around 8 and 16 hours postop, respectively. TnT levels were measured using a 4^th^ generation electrochemoluminescent enzyme immunoassay on an Elecsys 2010 platform (Roche Diagnostics, Mannheim, Germany). The lower detection limit of this assay is 0.01ng/L. CK levels were measured using a UV-test on a Roche Modular P800 (Roche Diagnostics), and CK-MB levels were measured using an electrochemoluminescent immunoassay (ECLIA) on a Roche Modular E170 platform (Roche Diagnostics).

Perioperative characteristics that were collected are listed in Tables 1 and 2. Suspicion of confounding was based on established associations with both main predictor and outcome measure, biological plausibility or inhomogeneous distributions at P-values < 0.1.

**Table 1:** Characteristics of 290 patients undergoing CABG.

		**without complication**	**with complication**	**p-Value**
		**(n=254)**	**(n=36)**	
Female (%)		43 (16.9%)	9 (25.0%)	.248
Age (years)		65.7 ± 9.7	66.5 ± 11.1	.400
Size (cm)		170.6 ± 9.4	170.2 ± 9.5	.603
Weight (kg)		81.5 ± 14.7	78.5 ± 17.7	.275
BSA (m^2^)		1.9 ± 0.1	1.8 ± 0.2	.390
BMI (kg/m^2^)		28 ± 4.7	26.9 ± 5	.188
Diabetes (%)		69 (27.2%)	11 (30.6%)	.692
Dyslipidemia (%)		207 (82.8%)	30 (83.3%)	1.000
Hypertension (%)		194 (76.4%)	31 (86.1%)	.284
Smoking (%)		150 (59.3%)	23 (65.7%)	.581
CV hereditary (%)		87 (37.2%)	12 (37.5%)	1.000
Serum creatinin (umol/L)		83.2 ± 23.5	107.6 ± 50.8	.006
Renal insufficiency (%)		6 (2.4%)	8 (22.2%)	<0.001
COPD (%)		25 (10.0%)	8 (23.5%)	.039
CCS class (%)	0	50 (19.7%)	4 (11.1%)	
	1	13 (5.1%)	1 (2.8%)	
	2	109 (42.9%)	15 (41.7%)	.362
	3	57 (22.4%)	9 (25.0%)	
	4	25 (9.8%)	7 (19.4%)	
NYHA class (%)	1	106 (41.7%)	9 (25.0%)	<0.001
	2	105 (41.3%)	12 (33.3%)	
	3	41 (16.1%)	12 (33.3%)	
	4	2 (.8%)	3 (8.3%)	
EuroSCORE additive		3.1 ± 2.3	5.2 ± 3	<0.001
EuroSCORE logistic		3 ± 2.6	6.7 ± 10.2	0.000
LVEF (%)		57.4 ± 12.2	50.1 ± 14.9	0.006
Urgency / Emergency (%)		42 (16.6%)	11 (30.6%)	0.063
Previous stroke (%)		10 (4.0%)	3 (8.6%)	0.205
Previous PCI (%)	0	202 (81.8%)	24 (68.6%)	0.155
	1	44 (17.8%)	11 (31.4%)	
	2	1 (.4%)	0 (.0%)	
Redo (%)		2 (.8%)	2 (5.6%)	0.770
PAOD (%)		10 (3.9%)	0 (.0%)	0.479
Carotid stenosis / previous carotid surgery (%)		22 (10.4%)	4 (12.9%)	0.754

CABG, coronary artery bypass grafting; BSA, body surface area, BMI, body mass index; COPD, chronic obstructive pulmonary disease; CCS, Canadian Cardiovascular Society; NYHA, New York Heart Association; LVEF, left ventricular ejection fraction; PCI, percutaneous coronary intervention; PAOD, peripheral arterial obstructive disease

**Table 2:** Coronary artery disease and procedure related factors in 290 patients undergoing CABG.

	**without complication**	**with complication**	**p-Value**
	**(n=254)**	**(n=36)**	
Left main (%)	33 (13.0%)	7 (19.4%)	0.303
IMA (%)	236 (92.9%)	35 (97.2%)	0.485
Operation duration (min)	199 ± 46.3	198.5 ± 55.2	0.769
Perfusion time (min)	73.2 ± 26	75.6 ± 30.5	0.935
X-clamp time (min)	47.5 ± 18.8	45.3 ± 14.6	0.643
CAD (number of vessels)	2.7 ± 0.4	2.8 ± 0.3	0.706
Distal anastomosis (n)	3.1 ± 0.8	3.2 ± 0.8	0.662
Defibrillation (%)	33 (13.0%)	7 (19.4%)	0.303

IMA, internal mammarian artery; CAD, coronary artery disease

### Data source, definitions and management of missing values

All variables were available from the prospective electronic registry, which includes follow-up data obtained systematically one year after surgery, then on a regular basis. Before analysis, data was scrutinized for completeness, plausibility and outliers by two data managers, who were independent of the current study. In case of missing or obviously incorrect values, alternative data sources such as hospital records were used for data replication.

Diagnosis of a perioperative MI was based on the recently published *Third Universal Definition of Myocardial Infarction* [16], and on the criteria used in the SYNTAX trial [17]. In brief, an MI was assumed if 2 or more out of a set of predefined criteria were met within the first 7 days after CABG (Table 3). Electrocardiograms (ECG) were all evaluated independently by a senior physician and a cardiologist, both of whom were blinded. Postoperative echocardiography was performed by a physician independent of the current study and only if clinically indicated.

**Table 3:** Diagnosis of perioperative myocardial infarction (MI).

Universal definition of MI (Type 5 MI)16	Definition of MI SYNTAX study ^17^	Definition of MI used in present study
Increase of biomarkers greater than 5x99th percentile upper reference limit during first 72 h following CABG; Pathological Q-waves or new left bundle-branch block, or angiographically documented new graft or native coronary artery occlusion, or imaging evidence of new loss of viable myocardium	***Withinthefirst7dayspostintervention* (*PCIorCABG*)**: Either new, abnormal Q waves and 1 ratio of peak creatine kinase–MB (CK-MB)/peak total CK >10% or new, abnormal Q-waves and 1 plasma level of CK-MB 5× upper limit for normal; ***7daysafteranyinterventionprocedure* (*PCIorCABG*)**: either new, abnormal Q waves or enzyme changes defined as more than 10% of the ratio of peak CK-MB/peak total CK on one or more than one sample (if no ratio is available—one or more than 1 plasma level of CK-MB 5× upper limit for normal)	2 or more of the 3 following criteria within the first 7 days following CABG:**1**. new Q-wave or new left bundle-branch block compared to the preoperative echocardiography (ECG); **2.** creatine kinase-MB (CK-MB) >60 mg/l; **3.** postoperative severe wall motion abnormalities in ECG corresponding with ischemic ECG changes or angiographically documented new graft or native coronary artery occlusion or myocardial necrosis diagnosed by autopsy

PCI, percutaneous coronary intervention; CABG, coronary artery bypass grafting

Postoperative new-onset renal insufficiency was defined by a new need for dialysis or, in patients with preoperative creatinin levels below 2mg/dl (172 µmol/L), if the postoperative creatinin level raised above 2mg/dl and reached at least double the preoperative value during hospitalization.

### Statistical analysis

Statistical analysis was performed by a biostatistician (BG) using SPSS for Windows (version 17.0; SPSS Inc, Chicago, IL, USA). Continuous variables are summarized as mean ± SD or, when skewed, as median and interquartile ranges. Non-parametric tests (Mann-Whitney) were used for statistical comparisons except for paired analyses comparing early and late TnT or CK-MB levels. Dichotomous variables are expressed in absolute numbers and percentages, and comparisons were made using a Chi square test. Multiple logistic regression modeling was used to assess independent associations between predictor and in-hospital complications. The TnT threshold value with the best predictive value was evaluated using ROC curve analysis. All tests were 2-sided and *P* values < 0.05 were assumed to indicate statistically significant differences.

## Results

**Figure 1:**
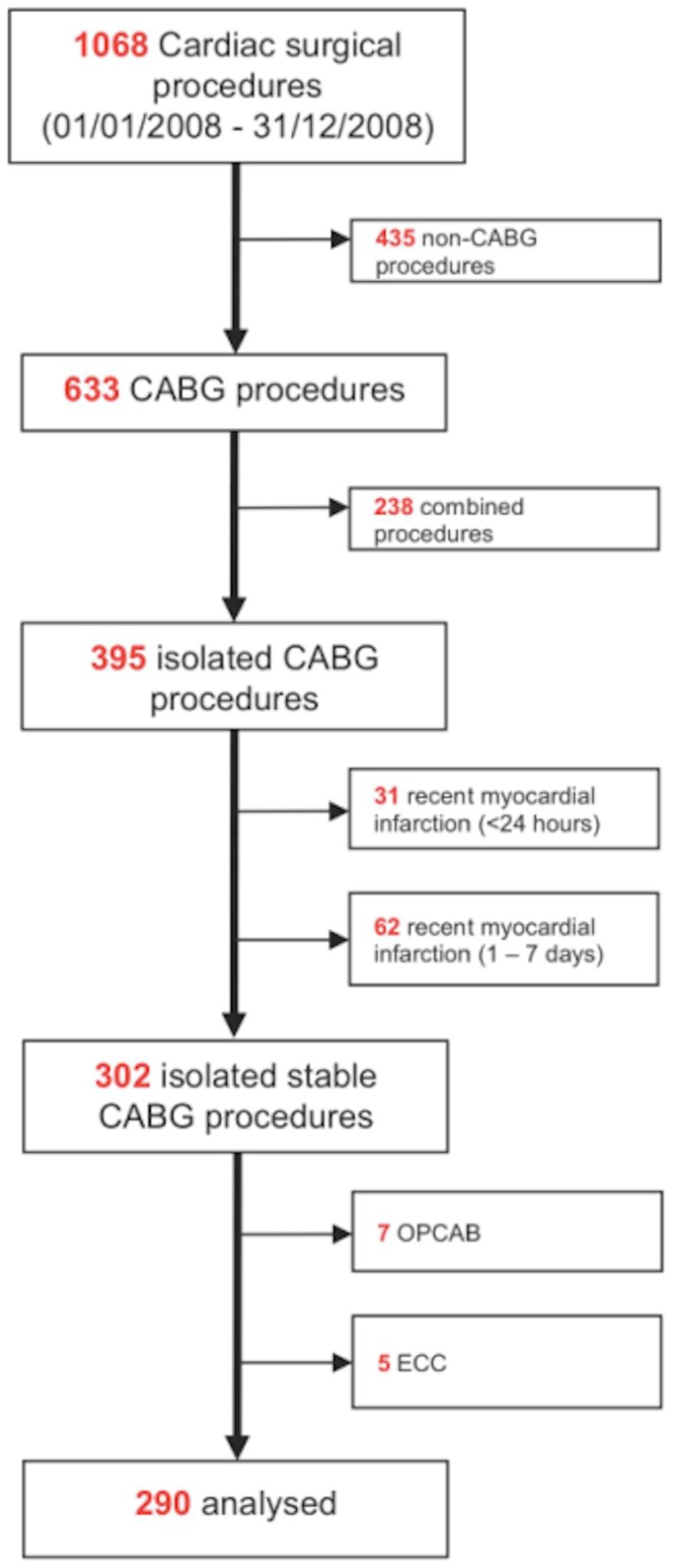
Selection process.

Out of 1068 consecutive patients who underwent cardiac surgery during the study period, 290 were included in the present investigation (Figure 1). Baseline characteristics of the study population are summarized in Table 1, and perioperative information is given in Table 2. Some 36 patients (12%) suffered a complication during hospitalization (Table 4): 6 had an MI, 4 a stroke, 8 an acute renal failure, 12 needed ICU support for longer than 48h, 4 were secondarily readmitted to the ICU for various reasons, 1 patient was resuscitated and 17 required vasopressors for more than 24 hours after surgery. One patient died on the 2^nd^ postoperative day and the autopsy confirmed an acute MI although all bypass grafts were open. Patients with or without complication differed regarding preoperative EuroSCORE, left ventricular ejection fraction (LVEF), and serum creatinin level (p<0.001 for all 3 parameters, Table 1). In contrast, cardiovascular risk factor profiles and severity of coronary artery disease (CAD) were similar between the groups, as were the proportion of left main CAD, the number of bypasses performed and the Canadian Cardiovascular Society (CCS) class. The duration of surgery as well as X-clamp and ECC times were also similar in both groups (Table 2).

**Table 4:** Postoperative course and distribution of complications in 290 patients undergoing CABG.

	**without complication**	**with complication**	**p-Value**
	**(n=254)**	**(n=36)**	
Reoperation	7 (2.8%)	3 (8.6%)	.108
ICU stay (days)	0.9 ± 0.2	2 ± 1.9	<0.001
ICU >2 days (%)	0	12 (33.3%)	<0.001
ICU readmission (%)	0	4 (11.1%)	<0.001
ICU stay total (days)	0.9 ± 0.2	2.8 ± 4.3	<0.001
Intubation time (days)	0.5 ± 0.1	1.1 ± 0.9	<0.001
Hosp duration (days)	7.1 ± 3.2	12.1 ± 9.1	<0.001
Mortality (%)	0	1 (2.8%)	.124
Stroke (%)	0	4 (11.1%)	<0.001
Post-operative creatinin (umol/L)	95.9 ± 39.9	177.4 ± 121.8	<0.001
New renal insufficiency (%)	0	8 (22.9%)	<0.001
New Dialysis (%)	0	1 (2.9%)	.121
Post-operative MI (%)	0	6 (16.7%)	<0.001
Resuscitation (%)	0	1 (2.8%)	.124
Atrial fibrillation (%)	44 (17.4%)	2 (6.3%)	.129
Need for blood products (%)	119 (47.6%)	20 (64.5%)	.088
Need for vasopressors at 6h (%)	24 (9.4%)	12 (33.3%)	<0.001
Need for vasopressors at 24h (%)	0	17 (47.2%)	<0.001
Adrenaline_6h (µg)	440 ± 211	630 ± 832	0.522
Dobutrex_6h (µg)	31.5 ± 22.1	50.1 ± 11.3	.061
Noradrenaline_6h (µg)	459 ± 245	1433 ± 2303	.518
Adrenaline_24h (µg)	92 ± 156	621 ± 1085	.354
Dobutrex_24h (µg)	71.4 ± 49.4	161.3 ± 158.9	.021
Noradrenaline_24h (µg)	266 ± 452	172 ± 203	.943

ICU, intensive care unit

As expected, peak TnT (1.1±1.4 ng/ml) as well as TnT values measured at 16 to 20 hours after surgery (1.0±1.4 ng/ml) were markedly increased in patients who suffered postoperative complications as compared to those who did not (0.31±0.28 ng/ml and 0.37±0.28 ng/ml, respectively, p<0.005). Although not as marked, differences were already significant at 6 to 8 hours after surgery (0.7±0.8 ng/ml versus 0.3±0.2 ng/ml, p = 0005; Table 5). A similar constellation was observed for CK-MB values (Table 5). Only 12% of the patients (n=36) required vasopressors during the first 6 postoperative hours; however, this was much more likely in patients who eventually developed a complication (33% versus 9%, p<0.001, Table 3).

**Table 5:** Assessment of early enzyme levels in 290 patients undergoing CABG.

**Laboratory**	**without complication**	**with complication**	**p-Value**
	**(n=254)**	**(n=36)**	
Delay before 1^st^ post-operative laboratory analysis (hours)	8.1 ± 1.0	8.1 ± 1	.554
CK_6 to 8h (U/l)	310.4 ± 168.9	396.9 ± 304.8	.356
CK-MB_6 to 8h (U/l)	13.2 ± 8.5	25 ± 27.2	.007
TnT_6 to 8h (ng/ml)	0.3 ± 0.2	0.7 ± 0.8	.005
Delay before 2^nd^ post-operative laboratory analysis (hours)	16.1 ± 2.4	17.3 ± 2.6	.027
CK_20h (U/l)	635.7 ± 638.8	859.1 ± 1231.6	.942
CK-MB_20h (U/l)	16.1 ± 15.8	34 ± 45.3	.051
TnT_20h (ng/ml)	0.3 ± 0.2	1.0 ± 1.4	<0.001
Max_CK (U/l)	634.9 ± 631.7	840 ± 1145.8	.757
Max_CK-MB (U/l)	17.3 ± 15.6	37.6 ± 44.7	.004
Max_TnT (ng/ml)	0.3 ± 0.2	1.1 ± 1.4	.002

CK, creatine kinase; TnT, troponin T

Multivariate analyses adjusted for two suspected confounding factors: the need for vasopressors during the first 6 postoperative hours and the logistic EuroSCORE which, in turn, is representative of a number of patient-related differences including NYHA class, pre-existing renal insufficiency, chronic lung disease, LVEF and urgency of the procedure. Both covariates correlated independently with the risk of developing a clinically relevant complication. Odds ratios (95% confidence intervals) for developing a complication were 6.82 (2.17, 21.40; P=0.001) for early TnT, 2.66 (1.00, 7.08; P=0.05) if vasopressors were needed during the first 6 postoperative hours, and 1.17 (1.05, 1.31; P=0.007) for logistic EuroSCORE, respectively. The overall R^2^ for this model was 0.359, the −2 log likelihood was 78.44 and the constant −4.68.

**Figure 2:**
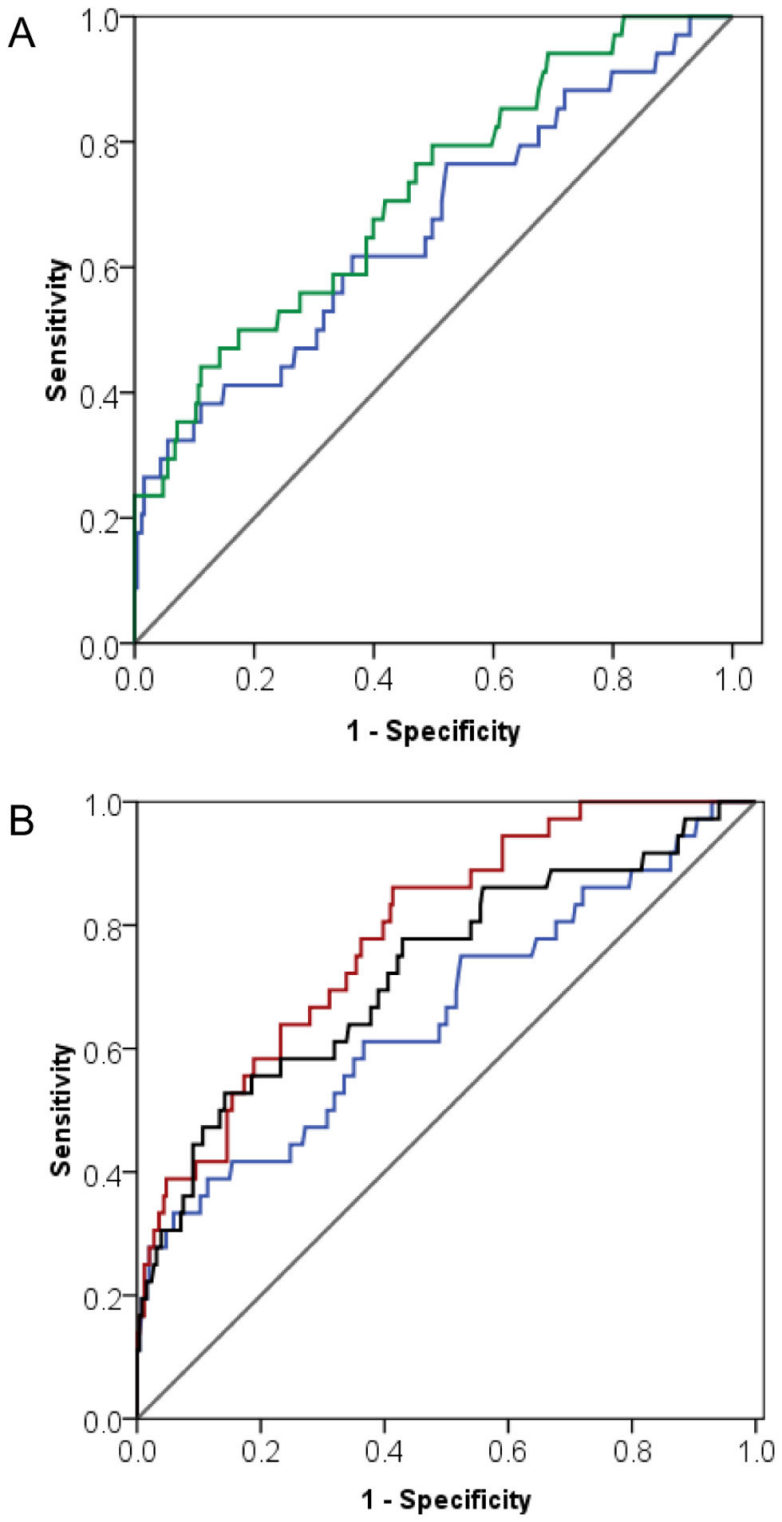
ROC curve analysis. A: ROC curves for TnT assessed at 6 to 8 hours post-surgery (AUC: 0.663; blue line) versus 20 hours (AUC: 0.717; green line). B: ROC curves for TnT assessed at 6 to 8 hours post-surgery when considered alone (AUC 0.663; blue line), together with the need of vasopressors during the first 6 hours post-surgery (AUC: 0.726; black line), and the preoperative EuroSCORE (AUC: 0.786 red line).

ROC curve analysis was used to determine an early TnT threshold with optimized sensitivity and specificity to predict postoperative complications (Figure 2 and Table 6). As expected, the area under the curve (AUC) was lower for TnT levels at 6 to 8 hours than for those at 20 hours (Figure 2A). However, in stratified analysis considering EuroSCORE and need for vasopressors, AUC increased considerably (Figure 2B). For instance, at a threshold level of 0.8 ng/ml, sensitivity increased from 0.28 to 0.44 if the early need for vasopressors was considered (Table 6).

**Table 6:** Stratified predictive characteristics of early TNT levels.

	All		Vasopressors within first 6 hours			
			No		Yes	
TnT	Spec	Sens	Spec	Sens	Spec	Sens
>1.1	.992	.194	.981	.167	1.000	.250
>1.0	.984	.222	.983	.208	1.000	.250
>0.9	.980	.222	.978	.208	1.000	.250
>0.8	.972	.278	.974	.208	.958	.417
>0.7	.949	.306	.957	.250	.875	.417
>0.6	.898	.361	.900	.333	.875	.417
>0.5	.839	.389	.843	.333	.792	.500
>0.4	.736	.444	.735	.417	.750	.500

Finally, early postoperative TnT levels were independently associated with long-term survival, which was assessed over a mean of 4 years (OR 4.0; CI: 2.09-7.72, p<0.001). 

## Discussion

Over the last decade, elective CABG has progressed to a very standardized and safe surgical procedure with low mortality and low rates of myocardial, neurologic, renal and other adverse events. Although influenced by the preoperative risk profile, the immediate postoperative course of these patients will depend on the surgical performance and perioperative factors. Thus, the present study focused on a potential indicator for risk stratification of intra- and postoperative course, and its findings suggest that early TnT levels, when elevated, independently predict the later occurrence of clinically relevant in-hospital complications after elective CABG procedures. The marker seemed particularly useful in high-risk patient subsets such as those with a high preoperative EuroSCORE or those requiring vasopressors during the initial postoperative period. Thus, the results of the current study, although based on the analysis of a restricted number of selected patients, suggest that early TnT elevations might be helpful to increase alertness for on-going, not yet detectable, myocardial complications and to guide early and intensified ICU management of CABG patients, with the further aim to reduce in-hospital complications and, more generally, to improve outcomes of CABG procedures.

Established cardiac risk scores such as the EuroSCORE are widely accepted predictors of postoperative complications and are useful to standardize comparisons. However, they do not account for the surgical intervention itself, although surgical difficulties, the duration of cross-clamping as well as the adequacy of myocardial protection will have an obvious influence on treatment success. Thus, complementary appraisal of procedural markers should facilitate a more precise risk stratification of patients admitted to the ICU after cardiac surgery. Early TnT levels represent a biologically plausible candidate for such a purpose, because they are cardiac specific and correlate directly with the number of damaged myocytes, thus reflecting myocardial injury that occurred during surgery [4].

This could help defuse the problem that during the earliest hours following cardiac surgery, on-going cardiac complications are often difficult to distinguish from transient hemodynamic instability due to the acute postoperative condition, the recovery from hypothermia and cardioplegia, the anesthesia or even the transport to the ICU. As a consequence, a myocardial cause of hemodynamic perturbations may be missed until perioperative MI has established itself irreversibly. The present study suggests that early TnT levels fulfill the criteria for a specific procedural marker within a useful therapeutic window because early TnT levels predicted outcome independently of the patients’ preoperative risk profile (i.e. the EuroSCORE) and independently of whether myocardial impairment was clinically relevant or silent (i.e. independently of any need for vasopressors). Interestingly, exposure to vasopressors during the initial 6 hours following surgery also predicted in-hospital outcome independently from the pre-operative EuroSCORE. Although these data confirm recently published results [18], they have to be validated in a larger study as well as in other centers, knowing there is no standardized protocol and thus a large variability among centers regarding the use of inotropes and vasopressors after cardiac surgery [19].

Clinical decision-making is generally complicated by the fact that TnT levels are almost always but inhomogeneously elevated after cardiac surgery. Therefore, no clear consensus has been reached thus far regarding timing of TnT measurements and definition of postoperative norms. Repetitive measurements have been suggested to determine the peak TnT level because of its good correlation with mid-term or even long-term outcome, which is likely based on its reflection of irreversible myocardial injury [3,6,9–12]. By comparison, the predictive value of early postoperative TnT levels is clearly inferior as shown by the present study as well as by others [5,10,13,14]. Nonetheless, according to the present data, early TnT levels correlate well enough with clinically important in-hospital complications to exceed the role of a descriptive prognostic factor into a useful diagnostic tool that could guide clinical management.

Clearly, even a robust correlation does not necessarily imply an absolute cut-off value, below which all cases take an uncomplicated course and vice versa. Instead, the predictive power increases with the pretest probability as seen in the present study when sensitivity of TnT increased notably in the presence of another independent predictor of complications, i.e. the need for vasopressors [18]. Thus, thresholds relate to a number of factors including the type of procedure, the patient population and the exact measurement timing [3,5,6,8,9,13]. The present study explored a relatively small but homogeneous population undergoing isolated CABG procedures under MECC [15], and evaluated a range of TnT measurements at 6 to 8 hours post-surgery. Thus, although present clinical outcomes fit well with other recently published results of CABG, generalization may be limited, and a causal relationship should not be inferred from this observational data. Not only the selected subgroup of cardiac surgery patients but also the limited study size and the low number of observed events may have impaired accuracy of ROC curve analyses. Therefore above all, the study represents a robust validation of a biologically plausible concept [3,5,6,8,9,13] and a basis for power calculations for larger scale studies that will be necessary to determine relative thresholds within specific clinical contexts. Another limitation relates to the fact that, due to the post-hoc study design, no interventions could be based on early TnT levels. The claim that early TnT levels may trigger clinical and cost effective measures to prevent complications from establishing is thus speculative and must be tested within a completely prospective study setting. Finally, new markers, such as the growth differentiation factor (GDF-15) or the heart-type fatty acid binding protein (hFABP), may provide even earlier information and with higher sensitivity [20,21] Further investigations as well as comparisons with more traditional approaches are, however, necessary.

## Conclusion

Although TnT is elevated in almost all patients immediately after CABG surgery, its early level may identify patients with an increased risk of developing clinically relevant in-hospital complications. The assumed correlation with myocardial injury was independent of both the preoperative patient risk profile and the immediate clinical symptoms. Therefore, early TnT level may become a promising complementary diagnostic for appraisal of intraoperative course and postoperative risk stratification. Its relative cut-off level and usefulness in guiding clinical decision-making need to be established in large-scale prospective studies.

## Supporting Information

Table S1 **Characteristics of 290 patients undergoing CABG.**
(DOCX)
