# Supplementary material for: Early Troponin T and Prediction of Potentially Correctable In-Hospital Complications after Coronary Artery Bypass Grafting Surgery
Source: PLoS One. 2013 Sep 6;8(9):e74241. doi: 10.1371/journal.pone.0074241 (PMC3765291; doi:10.1371/journal.pone.0074241)
Supplement: Table S1 — Characteristics of 290 patients undergoing CABG. (DOCX) [file pone.0074241.s001.docx]

**SUPPORTING INFORMATION**

**Table S1:** Characteristics of 290 patients undergoing CABG

|  | | **without complication** | **with complication** | **p-Value** |
| --- | --- | --- | --- | --- |
|  | | **(n=254)** | **(n=36)** |  |
| Female (%) | | 43 (16.9%) | 9 (25.0%) | .248 |
| Age (years) | | 65.7 ± 9.7 | 66.5 ± 11.1 | .400 |
| Size (cm) | | 170.6 ± 9.4 | 170.2 ± 9.5 | .603 |
| Weight (kg) | | 81.5 ± 14.7 | 78.5 ± 17.7 | .275 |
| BSA (m^2^) | | 1.9 ± 0.1 | 1.8 ± 0.2 | .390 |
| BMI (kg/m^2^) | | 28 ± 4.7 | 26.9 ± 5 | .188 |
| Diabetes (%) | | 69 (27.2%) | 11 (30.6%) | .692 |
| Dyslipidemia (%) | | 207 (82.8%) | 30 (83.3%) | 1.000 |
| Hypertension (%) | | 194 (76.4%) | 31 (86.1%) | .284 |
| Smoking (%) | | 150 (59.3%) | 23 (65.7%) | .581 |
| CV hereditary (%) | | 87 (37.2%) | 12 (37.5%) | 1.000 |
| Serum creatinin (umol/L) | | 83.2 ± 23.5 | 107.6 ± 50.8 | .006 |
| Renal insufficiency (%) | | 6 (2.4%) | 8 (22.2%) | <0.001 |
| COPD (%) |  | 25 (10.0%) | 8 (23.5%) | .039 |
| CCS class (%) | 0 | 50 (19.7%) | 4 (11.1%) |  |
|  | 1 | 13 (5.1%) | 1 (2.8%) |  |
|  | 2 | 109 (42.9%) | 15 (41.7%) | .362 |
|  | 3 | 57 (22.4%) | 9 (25.0%) |  |
|  | 4 | 25 (9.8%) | 7 (19.4%) |  |
| NYHA class (%) | 1 | 106 (41.7%) | 9 (25.0%) | <0.001 |
|  | 2 | 105 (41.3%) | 12 (33.3%) |  |
|  | 3 | 41 (16.1%) | 12 (33.3%) |  |
|  | 4 | 2 (.8%) | 3 (8.3%) |  |
| EuroSCORE additive | | 3.1 ± 2.3 | 5.2 ± 3 | <0.001 |
| EuroSCORE logistic | | 3 ± 2.6 | 6.7 ± 10.2 | 0.000 |
| LVEF (%) | | 57.4 ± 12.2 | 50.1 ± 14.9 | 0.006 |
| Urgency / Emergency (%) | | 42 (16.6%) | 11 (30.6%) | 0.063 |
| Previous stroke (%) | | 10 (4.0%) | 3 (8.6%) | 0.205 |
| Previous PCI (%) | 0 | 202 (81.8%) | 24 (68.6%) | 0.155 |
|  | 1 | 44 (17.8%) | 11 (31.4%) |  |
|  | 2 | 1 (.4%) | 0 (.0%) |  |
| Redo (%) | | 2 (.8%) | 2 (5.6%) | 0.770 |
| PAOD (%) | | 10 (3.9%) | 0 (.0%) | 0.479 |
| Carotid stenosis / previous carotid surgery (%) | | 22 (10.4%) | 4 (12.9%) | 0.754 |

CABG, coronary artery bypass grafting; BSA, body surface area, BMI, body mass index; COPD, chronic obstructive pulmonary disease; CCS, Canadian Cardiovascular Society; NYHA, New York Heart Association; LVEF, left ventricular ejection fraction; PCI, percutaneous coronary intervention; PAOD, peripheral arterial obstructive disease
